# Ketamine Inhalation Alters Behavior and Lower Urinary Tract Function in Mice

**DOI:** 10.3390/biomedicines11010075

**Published:** 2022-12-28

**Authors:** Shu-Yu Wu, Chun-Kai Hsu, Li-Yi Lim, Yi-Chyan Chen, Hsi-Hsien Chang, Stephen Shei-Dei Yang

**Affiliations:** 1 Department of Urology, Taipei Tzu Chi Hospital, Buddhist Tzu Chi Medical Foundation, New Taipei City 23142, Taiwan; 2 School of Medicine, Tzu Chi University, Hualien 97004, Taiwan; 3 Department of Surgery, Hospital Canselor Tuanku Muhriz UKM, Kuala Lumpur 56000, Malaysia; 4 Department of Psychiatry, Taipei Tzu Chi Hospital, Buddhist Tzu Chi Medical Foundation, New Taipei City 23142, Taiwan

**Keywords:** ketamine, ketamine inhalation, ultrasonic nebulizer, ketamine-induced uropathy

## Abstract

We aimed to evaluate behavioral and lower urinary tract changes in mice using a novel ketamine inhalation model mimicking human ketamine abusers and compare the results to those obtained using a ketamine intraperitoneal injection model. C57BL/6N mice were placed in a transparent acrylic observation cage connected to an ultrasonic nebulizer producing ketamine (KI) or saline (SI) fog. The mice were given KI or SI fog twice a week for three months. In another experiment arm, the mice were given intraperitoneal ketamine injections (KP) or saline injections (SP) twice a week for three months. The presence of urine ketamine (>100 ng/mL) was determined using a quick test kit. Locomotor activity was recorded by video using the open field test. Lower urinary tract function was assessed using urine spots, cystometry and histology. KI and KP mice crossed the center more frequently and traveled farther than SI and SP mice. Only KI mice, however, demonstrated popcorn-like jumping, and frequent center crossing. Detrusor overactivity, reduced cystometric bladder capacity, and denuded mucosa were observed in both KI and KP mice. Ketamine inhalation induces behavioral and lower urinary tract changes in mice that are comparable to intraperitoneal ketamine injections.

## 1. Introduction

Ketamine is a dissociative anesthetic agent. It is a Class III illicit substance in Taiwan, and 84% of users are men [1]. Ketamine has long been one of the most abused illegal drugs due to its analgesic, central excitatory, and hallucinogenic effects. Long-term recreational use can lead to psychological tolerance and dependence, resulting in unique pathological and psychological symptoms that are similar to schizophrenia [2,3]. Previous human and animal studies have shown that ketamine abuse not only causes cardiotoxicity [4] and neurotoxicity [5], but also has an adverse effect on the urinary system [6,7,8,9,10].

Traditionally, intraperitoneal injection was used in animal experiments to study the effects of ketamine [11,12,13,14]. However, the most common ways in which ketamine is abused are through nasal inhalation, nebulization, or mixing it with cigarettes. We created a ketamine inhalation model to accurately represent how the drug is abused. To improve ketamine transport across the nasal membrane, we administered ketamine fog to mice using an ultrasonic nebulizer in an airtight chamber. Following that, we compared the behavioral and urinary tract changes in mice after ketamine inhalation to those after intraperitoneal injection.

## 2. Materials and Methods

### 2.1. Ketamine Inhalation and Intraperitoneal Injection Models

**Figure 1:**
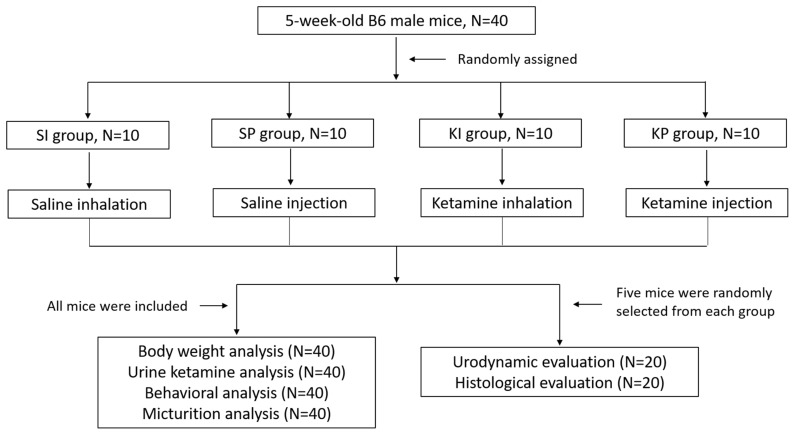
Flowchart of case distribution and interventions.

This experiment was approved by our institute’s Laboratory Animal Care and Use Committee. Four-week-old C57BL/6 male mice (B6) were maintained under controlled light (12 h light/dark cycles from 7:00 a.m. to 7:00 p.m.) and temperature (21 °C to 23 °C). Food and water were available ad libitum. One week later, these mice were subjected to experiments. The detailed case distribution and interventions are presented in Figure 1. 

The 5-week-old male mice were randomly assigned to saline or ketamine groups. For three months, five mice as a group were placed in an airtight whole-body exposure chamber for 45 min twice a week and exposed to 6 mL of normal saline (SI group, *n* = 10) or ketamine (KI group, *n* = 10) fog produced by a nebulizer (ULTRA-NEB^®^99, DeVilbiss Healthcare Co., Somerset, PA, USA). During the first month, the KI group received an initial dose of 100 mg of ketamine (2 mL of Ketalar 50 mg/mL injection, Pfizer) diluted with 4 mL of sterile normal saline). The dose was increased to 150 mg in the second month (3 mL of 50 mg/mL Ketalar diluted with 3 mL of sterile normal saline) and 300 mg in the third month (6 mL of 50 mg/mL Ketalar). In another experiment arm, 5-week-old mice were given an intraperitoneal injection of 25 mg/kg ketamine (Ketalar 50 mg/mL injection, Pfizer) twice a week for three months to mimic traditional animal studies (KP group, *n* = 10), while a control group of mice received an injection of saline solution (0.02 mL) (SP group, *n* = 10).

### 2.2. Urine Ketamine Qualitative Analysis and Body Weight

At three months, the urine ketamine qualitative tests were performed using a rapid test kit (Easy One Step Test. D014-C, Firstep Bioresearch, Inc., Tainan, Taiwan). This type of kit has been used by Taiwanese authorities to detect the presence of urine ketamine in suspected ketamine abusers. If the control line appears on the test cassette, it means the ketamine concentration in the urine is greater than 100 ng/mL. The result is negative when both the control and test lines are visible, indicating the absence or low level of ketamine in the urine. The mice were weighed once a week for three months.

### 2.3. Quantification of the Voiding Pattern

Voiding pattern assessments were carried out in all mice on a weekly basis. Voiding pattern measurements were taken 24 h before and after each ketamine/saline administration. Subjects were placed individually in cages with filter paper on the floor for 90 min. Filter papers were dried, exposed to ultraviolet light, and then photographed.

### 2.4. Behavioral Observation

To assess general locomotor activity, an open field test (OFT) with a video-tracking system (SINGA, TW) recording was performed for 5 min an hour before and 30 min after ketamine or saline administration. To avoid interference, OFTs were performed inside a Plexiglas open-view box (35 cm × 35 cm × 30 cm) illuminated with a 20-watt light bulb. The open-view box is open at the top and surrounded by a 1 cm thick wall. The square in the middle of the box is referred to as “centers”, while the areas outside of the square and adjacent to the wall are referred to as “peripheries”. (Figure S1). The mouse was placed in a random corner of the box. We use software (Singa Trace Mouse II) to calculate the total distance traveled, time spent in the center, and frequency of center crossings. Other information, such as the time spent before entering the center, frequency of defecation, and frequency of feeding, were also recorded.

### 2.5. Urodynamic Study (Cystometry)

In vivo cystometry was performed at the end of the 3-month period for both the saline and ketamine groups. Five mice were randomly selected from each group for urodynamic evaluation. Urethane (500 mg/kg) and chloralose (50 mg/kg) were used to sedate the mice. A lower abdominal incision was made, and a saline-filled polyethylene (PE) 10 catheter with a blunted end was sutured to the bladder dome with 6-0 sutures. The exteriorized cannula has two ports, one of which is connected to a pressure transducer and the other to a syringe pump (1.8 mL/h, 37 °C). Muscle and skin were closed separately using nonabsorbable sutures. The urinary bladder pressure was recorded on a Powerlab polygraph (ADInstruments Pty Ltd., Castle Hill, Australia).

### 2.6. Histology of Bladder Tissue

At the end of the study, five mice were randomly selected from each group for histological analysis. The bladder tissues were harvested after the mice were sacrificed. The tissues were fixed overnight in 4% paraformaldehyde with 0.1 M phosphate buffer at pH 7.4 and 4 °C. After rinsing in 0.1 M phosphate buffer, the specimens were dehydrated in ethanol, cleared in xylene, and embedded in paraffin. Each specimen was cut into 3 mm thick serial paraffin sections and stained with hematoxylin and eosin.

### 2.7. Statistical Analysis

A *t*-test was used to compare the differences between the control and ketamine-treated groups. An ANOVA of variance followed by post hoc tests (Bonferroni) was used to compare differences between different groups. All values are presented as mean ± SD. A *p*-value < 0.05 was considered statistically significant.

## 3. Results

### 3.1. Urine Ketamine Qualitative Analysis and Body Weight Changes

**Figure 2:**
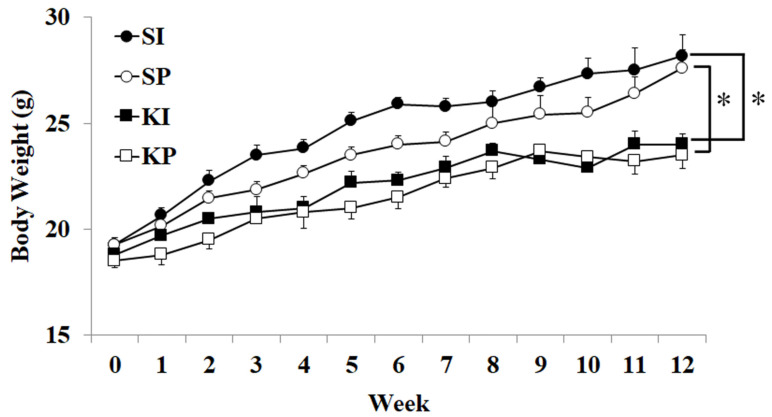
**Ketamine inhalation and body weight change.** KI and KP mice had lower body weights than SI and SP mice from the fourth weeks onward (*p* < 0.05). The values are expressed in means ± SD. An asterisk indicates a significant difference in Bonferroni post-tests following two-way ANOVA (*p* < 0.05).

Urine qualitative tests were positive in both KI and KP mice but negative in SI and SP mice (Figure S2). The body weights of KI and KP mice were significantly lower than those of SI and SP mice (Figure 2, *p* < 0.05).

### 3.2. Behavioral Changes after Ketamine Exposure

**Figure 3:**
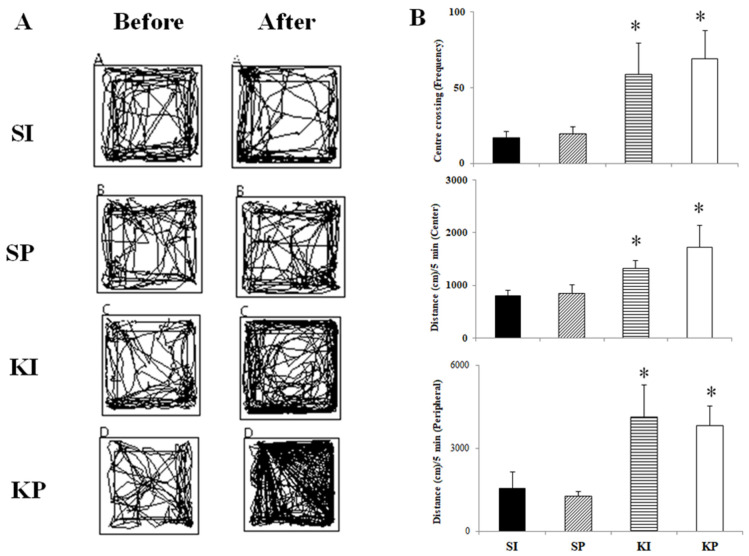
**The effect of ketamine inhalation on behavior during the open field test (OFT).** KI and KP mice had increased locomotor activity tracks, but SI and SP mice did not (**A**). KI and KP mice had higher center crossing frequency, center travel distance, and peripheral distance when compared to SI and SP mice (*p* < 0.05) (**B**). The values are expressed in means ± SD. An asterisk indicates a significant difference in Bonferroni post-tests following one-way ANOVA (*p* < 0.05).

Ketamine-induced hyperlocomotion was observed in both KI and KP mice following their first ketamine exposure (Figure 3A). KI and KP mice significantly increased their frequency of center crossing (Figure 3B, *p* < 0.001) and distance traveled (center and peripheral) in the OFT (Figure 3B, *p* < 0.001) when compared to their baseline before ketamine treatment as well as the results from the SI and SP groups. Popcorn-like jumping movements (Video S1), on the other hand, were only seen in KI mice and not in SI, KP, or SP mice.

### 3.3. The Effects of Ketamine Administration on Lower Urinary Tract Function

**Figure 4:**
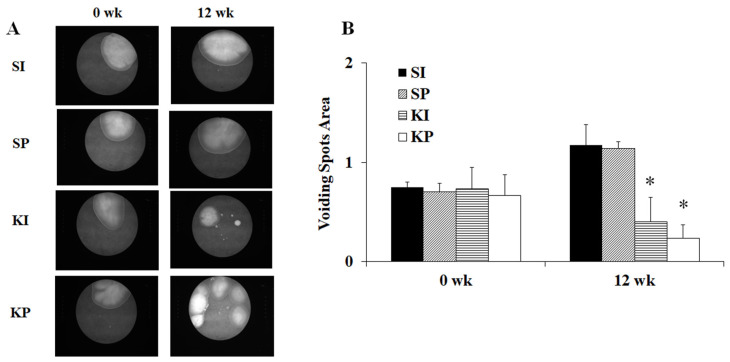
**The effect of ketamine on micturition.** When compared to SI and SP mice, KI and KP mice had more urine spots but smaller urine spots size (**A**). The voiding spots area of KI and KP mice had significantly decreased after 12 weeks of ketamine treatment (*p* < 0.05) (**B**). The values are expressed in means ± SD. An asterisk indicates a significant difference in Bonferroni post-tests following one-way ANOVA (*p* < 0.05).

When compared to SI and SP mice, KI and KP mice had more urine spots but smaller urine spots size, indicating an increase in voiding frequency but a decrease in voided volume (Figure 4A). KI and KP mice had significantly smaller mean urine spot areas than SI and SP mice (Figure 4B, *p* < 0.05).

**Figure 5:**
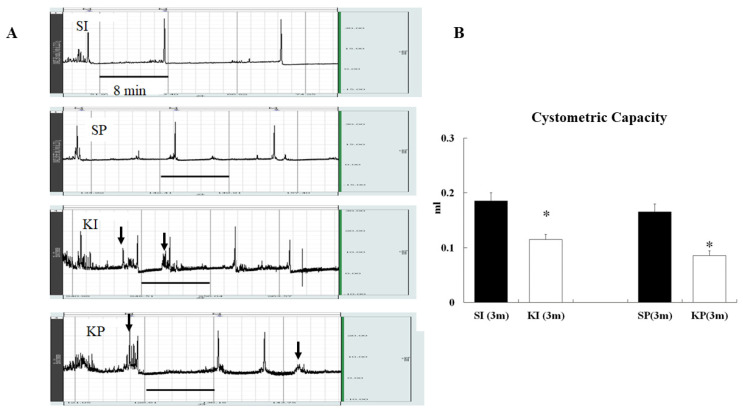
**Ketamine inhalation and urodynamic changes.** Unstable detrusor contractions (black arrows) were recorded in the cystometry curves of KI and KP mice but not in SI and SP mice (**A**). At 3 months, KI and KP mice had significantly lower bladder capacity than SI and SP mice (*p* < 0.05) (**B**). The values are expressed in means ± SD. An asterisk indicates a significant difference in Bonferroni post-tests following *t*-test (*p* < 0.05).

Cytometry of KI and KP mice after 3 months of ketamine exposure revealed numerous non-micturition-related detrusor contractions indicative of detrusor overactivity (DO) (Figure 5A). Furthermore, the mean cystometric capacity of the KI mice was significantly lower than that of the SI mice (Figure 5B, *p* < 0.05). There were no significant changes in cystometric capacity and no evidence of DO in SI and SP mice.

**Figure 6:**
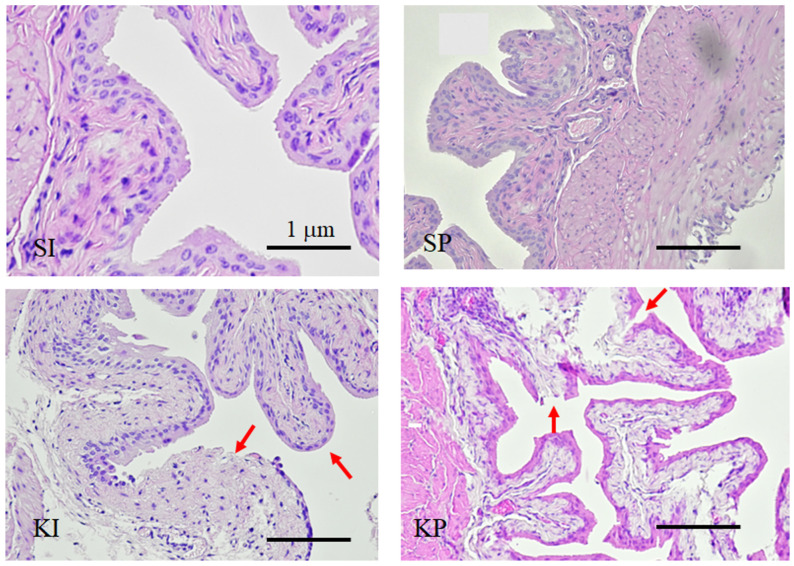
**Ketamine inhalation and histological changes.** Denuded urothelium (arrows) with subcostal edema were observed only in KI and KP mice but not in SI and SP mice.

Significant histopathological changes were found in the urinary bladders of KI and KP mice. At the end of three months, both KI and KP mice exhibited a smooth apical epithelial surface, urothelial denudation with submucosal congestion, and edema (Figure 6), while the urothelium of both SI and SP remained intact. Table 1 summarizes the above results.

## 4. Discussion

This is the first study to show behavioral change and lower urinary tract dysfunction in mice using a ketamine inhalation model that mimics human behavior. Ketamine was found in the urine of KI and KP mice but not in the urine of SI and SP mice (Figure S2). The effects of ketamine on the bladder can be caused by both ketamine and its metabolites in urine [15]. Furthermore, urinary ketamine and ketamine metabolites were found to be positively correlated with the degree of overactive bladder and the numeric pain rating scale in ketamine abusers [16]. In the current study, ketamine inhalation resulted in weight loss, behavioral changes, and bladder dysfunction.

Reduced body weight gain was statistically lower in KI and KP mice than in SI and SP mice (Figure 2). Body weight loss has also been reported in human ketamine abusers [17,18]. Body weight loss and altered voiding habits in ketamine-abusing male mice were positively correlated with elevated levels of ketamine and norketamine detected in the urine [19]. Reduced body weight may be a result of the physiological stress caused by ketamine administration [19].

Increased center exploration (Figure 3A) and longer travel distance (Figure 3B) in the OFT were observed in both KI and KP mice but not in SI or SP mice. Previous research had shown that noncompetitive NMDA antagonists such as MK-801, phencyclidine, and ketamine increased locomotion in rodents [20,21]. The noncompetitive NMDA antagonists may elicit locomotion by enhancing glutamatergic transmission within the mesolimbic dopamine system [22]. Our findings echoed the results of Hetzler BE et al. that ketamine-induced locomotion consisted primarily of ambulation around the perimeter of the field and was accompanied by ataxia [12]. Popcorn-like jumping movements (Video S1) and increased center crossing after ketamine inhalation were observed only in KI mice. Similar jumping behavior had been observed in rodents treated with MK-801 (NMDA receptor antagonist) and phencyclidine (noncompetitive NMDA receptor antagonist) [23,24]. Ketamine inhalation did induce similar behavioral changes secondary to the intraperitoneal injection model and unique behavioral changes only observed in KI mice.

Increased voiding frequency with decreased voided volume was observed in both KI and KP mice (Figure 4) as well as in human ketamine abusers [17,18]. Furthermore, decreased cystometric capacities linked to DO were observed in both KI and KP mice (Figure 5). It is well known that ketamine causes urinary tract dysfunction in mice, including overactive or underactive bladders [25,26], reduced bladder capacity [18], and altered contractility [27]. Furthermore, both KI and KP mice displayed the typical changes of ketamine-induced cytopathy, including a smooth apical epithelial surface, urothelial denudation with submucosal congestion, and edema (Figure 6). This study discovered that ketamine inhalation and intraperitoneal injection both damage lower urinary tract function and histopathology in a similar way.

The first limitation of this study is that we only looked at the effects of ketamine in male mice. Given that the majority of ketamine users are men, this approach is reasonable. Women who abuse ketamine, however, appear to have more severe lower urinary tract symptoms and/or dysfunction [1,28]. Further research into the impact of ketamine abuse on different genders should be encouraged. The second limitation is that we did not assess the serum ketamine level following ketamine administration, though we did demonstrate that ketamine was present in urine following ketamine administration using a urine test kit that is employed by law enforcement. We understand that the actual dose of ketamine inhaled may vary, but this accurately reflects the real-world situation in which determining the actual amount of ketamine use is difficult. The third limitation is that we did not investigate the dose–effect relationship of ketamine on behavioral and urinary tract function. Pharmacodynamic and pharmacokinetic studies of ketamine inhalation at various doses, frequencies, and durations should be the focus of future research.

## 5. Conclusions

This ketamine inhalation model mimics human drug use, and it demonstrated that ketamine inhalation in mice resulted in behavioral changes and lower urinary tract dysfunction, which are similar to the conventional ketamine intraperitoneal injection model and human ketamine abuse. The ketamine inhalation model is more animal-friendly since it eliminates the need for multiple peritoneal injections. More research is needed to validate the effects of this novel animal experimentation model.

## Figures and Tables

**Table 1:** Summary of the results.

	SI Group	SP Group	KI Group	KP Group	*p*-Value
Body weight (g)	28.2 ± 1.7	27.6 ± 1.4	24.0 ± 1.9	24.0 ± 1.4	0.03
Urine ketamine (ng/mL)	Negative	Negative	Positive	Positive	
Open field test (cm)	798.4 ± 110.1	908.0 ± 165	1331.1 ± 134.1	1728.3 ± 409.4	0.01
Urine spots size (cm^2^)	1.17 ± 0.21	1.14 ± 0.07	0.40 ± 0.25	0.24 ± 0.13	0.02
Cystometric capacity (mL)	0.17 ± 0.01	0.15 ± 0.01	0.12 ± 0.01	0.09 ± 0.01	0.01

SI: saline inhalation, SP: intraperitoneal saline injection, KI: ketamine inhalation, KP: intraperitoneal ketamine injection.

## Data Availability

Data is available on request to the corresponding author.

## Supplementary Materials

The following supporting information can be downloaded at: https://www.mdpi.com/article/10.3390/biomedicines11010075/s1, Figure S1: The center and periphery zones in the OFT test; Figure S2: Results of the ketamine rapid test; Video S1: Popcorn-like jumping was recorded after ketamine inhalation.
