# Supplementary material for: Ketamine Inhalation Alters Behavior and Lower Urinary Tract Function in Mice
Source: Biomedicines. 2022 Dec 28;11(1):75. doi: 10.3390/biomedicines11010075 (PMC9855675; doi:10.3390/biomedicines11010075)
Supplement: Supplementary file 1 [file biomedicines-11-00075-s001.zip › biomedicines-2058521-supplementary.pdf]

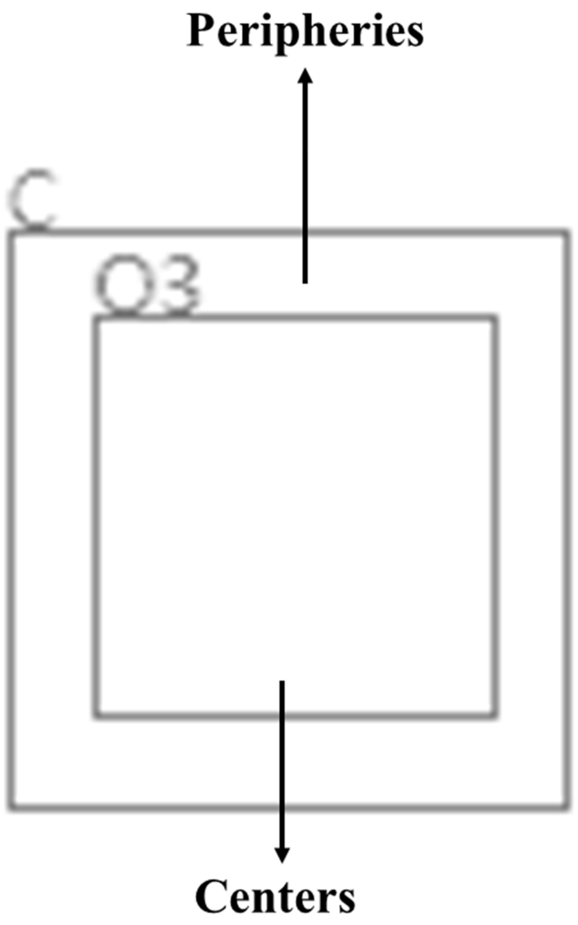

Figure S1. The center and periphery zones in the OFT test.

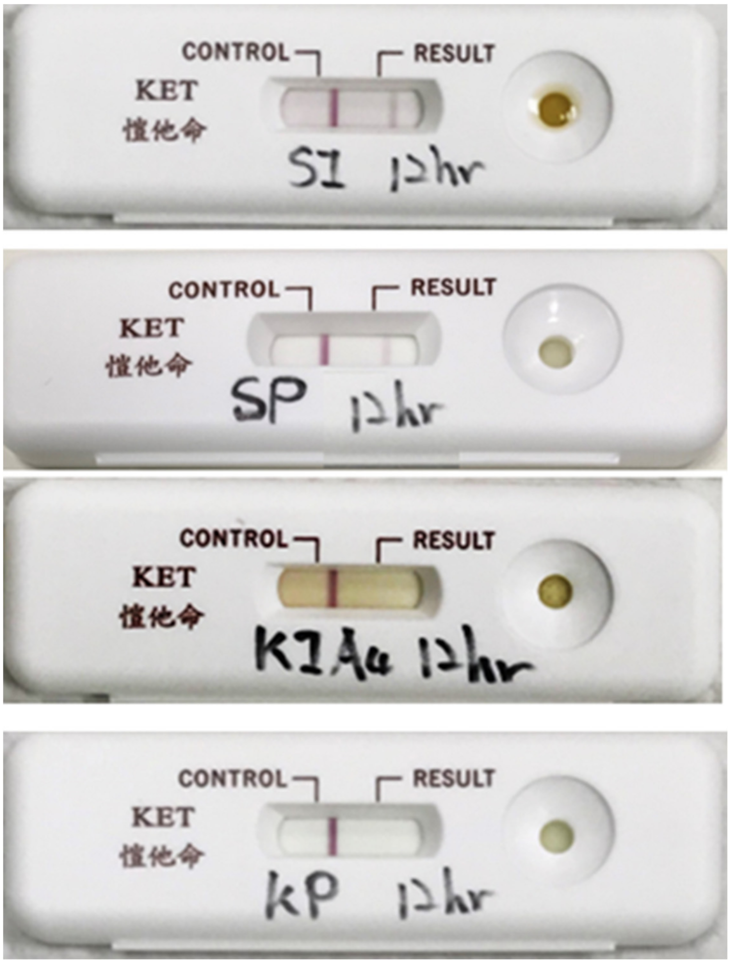

Figure S2. Results of the ketamine rapid test.
